# FAM83A Promotes the Proliferative and Invasive Abilities of Cervical Cancer Cells via Epithelial-Mesenchymal Transition and the Wnt Signaling Pathway

**DOI:** 10.7150/jca.62563

**Published:** 2021-08-28

**Authors:** Chong Lan, Chen-Chen Liu, Xiao-Cui Nie, Lei Lei, Zhang-Xian Xiao, Ming-Xi Li, Xue-Nan Tang, Ming-Yu Jia, Hong-Tao Xu

**Affiliations:** 1 Department of Gynecology, Shenyang Women and Children's Hospital, Shenyang, China.; 2 Department of Pathology, the First Hospital and College of Basic Medical Sciences, China Medical University, Shenyang, 110001, China.

**Keywords:** Family with sequence similarity 83, Cervical neoplasm, Squamous cell carcinoma, Wnt signaling pathway, Epithelial-mesenchymal transition

## Abstract

The family with sequence similarity 83, member A (*FAM83A*) gene is associated with the occurrence and development of many malignant tumors. Our aim was to explore the role of FAM83A in cervical cancer. FAM83A was overexpressed or knocked down in cervical cancer cells, and the expressions of FAM83A, key proteins involved in the epithelial-mesenchymal transition (EMT), and Wnt signaling pathway-related proteins were detected by western blot analysis. Cell proliferative and invasive abilities were also examined using cell proliferation, colony formation, and Matrigel invasion assays. Cells were treated with the Wnt pathway inhibitor XAV-939 to determine whether Wnt signaling was necessary for the effect of FAM83A on cervical cancer cells. FAM83A was highly expressed in cervical cancer tissues and was associated with differentiation, TNM stage, lymph node metastasis, and poor prognosis in patients with cervical cancer. Knockdown of FAM83A inhibited the proliferation, colony formation, and invasion of cervical cancer cells. The opposite results were observed in FAM83A-overexpressing cells, and FAM83A overexpression also promoted EMT and Wnt signaling. XAV-939 reversed the activation of Wnt signaling and EMT induced by FAM83A. In conclusion, FAM83A expression was increased in cervical cancers and correlated with poor prognosis of patients. FAM83A overexpression can activate the Wnt signaling pathway, facilitate EMT, and promote the proliferative and invasive abilities of cervical cancer cells.

## Introduction

Cervical cancer is the fourth most frequently diagnosed cancer and the fourth leading cause of cancer-related death in women. In 2008, there were more than half a million new cases and 300,000 deaths worldwide 1, 2. Presently, human papillomavirus vaccination and cervical cancer screening are performed to prevent cervical cancer, and surgery, chemotherapy, and radiotherapy are used to treat it. However, these treatment modalities are associated with severe side effects, and once disease recurrence occurs, the prognosis is relatively poor because of limited clinical therapies 3, 4.

The family with sequence similarity 83, member A (*FAM83A*) gene, also called BJ-TSA-9, is the smallest of the eight members of the FAM83 family 5. The family members share a highly conserved N-terminal domain (DUF1669 domain), which is thought to be involved in tumor progression 6, whereas the C-terminal regions vastly differ between the different family members 7. *FAM83A* was originally identified as a tumor-specific gene by a bioinformatics approach in 2005 8. FAM83A is considered to be involved in the progression of many human cancers and is associated with poor prognosis. FAM83A promotes tumor progression and increases proliferation, invasion, drug resistance, and stem cell-like traits in breast, lung, hepatocellular, and pancreatic cancers 5, 9-12. A recent study indicated that FAM83A is regulated by miR-206 to promote cervical cancer development via the PI3K/AKT/mTOR pathway 13. Xu et al. also reported that FAM83A was associated with cervical lesion progression and carcinogenesis and that it was specific for cervical cancer as opposed to cervical intraepithelial neoplasia (CIN) 2 to CIN 3 and normal cervical epithelium 14. However, the same authors recently reported that FAM83A plays an anticancer role in cervical cancer by regulating integrins 15. Therefore, the role of FAM83A in cervical cancer needs to be clarified and confirmed.

Chen et al. reported that aberrant amplification of FAM83A promotes pancreatic cancer stem cell-like traits by activating the TGF-β and Wnt signaling pathways, resulting in poorer clinical outcomes in patients with cancer 9. The Wnt signaling pathway is essential in both normal embryonic development and in the carcinogenesis of various cancers 10, 16-18.

Local infiltration and distant metastasis are the main biological characteristics of malignant tumors. Epithelial-mesenchymal transition (EMT) can reduce adhesions between cells, leading to tumor progression and distant metastasis 19, 20. Zhou et al. first highlighted the role of FAM83A in promoting EMT and metastasis in non-small cell lung cancer 5. However, whether FAM83A regulates the Wnt signaling pathway and EMT in cervical squamous cell carcinoma (CSCC) is unclear.

This study examined the expression of FAM83A and its effects on the proliferation and invasion of CSCC cells and explored the underlying mechanisms of FAM83A-mediated regulation of EMT and the Wnt signaling pathway.

## Materials and methods

### Online data collection and analysis

The expression of FAM83A in cervical cancers and normal cervical tissues and the correlation of the expression of FAM83A with cervical cancer outcomes were retrieved and downloaded from UALCAN (http://ualcan.path.uab.edu) 21 and cBioPortal (http://www.cbioportal.org) 22, which are web-based tools for data mining based on The Cancer Genome Atlas (TCGA) and Genotype Tissue Expression data. Survival curves for patients with cervical cancer with high or low FAM83A expression were obtained using the Kaplan‑Meier plotter (http://kmplot.com/analysis/) 23.

### Patients and specimens

Total of 60 CSCC specimens were collected from the patients (average age of 60 years) who underwent complete surgical resection at the First Affiliated Hospital of China Medical University between 2018 and 2019. Some of the CSCC samples were accompanied by corresponding normal cervical tissue samples. All patients did not undergo chemotherapy and radiotherapy prior to resection. The CSCCs were classified as well (19 cases), and moderately-poorly differentiated (41 cases). According to the seventh edition of the International Union against Cancer TNM classification of carcinomas of the uterine cervix, patients were categorized as stage I (n = 14), II (n = 23), or III (n = 23). Lymph node metastases were observed in 21 patients. Written informed consent was obtained from all the patients, and all procedures were approved by the Research Ethics Committees of China Medical University and Shenyang Women and Children's Hospital.

### Immunohistochemistry

Briefly, tissue sections of 4 μm were deparaffinized in xylene, rehydrated in a gradient alcohol series, and boiled in 0.01 M citrate buffer (pH 6.0) for 2 min in an autoclave. Endogenous peroxidase activity was blocked using hydrogen peroxide (0.3%), which was followed by incubation with normal goat serum to reduce nonspecific binding. The sections incubated with an anti-FAM83A rabbit polyclonal antibody (1:200, bs-16014R, Beijing Biosynthesis Biotechnology, China). The intensity of staining was scored as follows: 0, no staining; 1 weak; 2, moderate; and 3, high. Percentage scores were categorized as follows: 1 (1%-25%), 2 (26%-50%), 3 (51%-75%), and 4 (76%-100%). The scores for each tumor sample were multiplied to obtain a final score ranging from 0 to 12. Tumor samples with scores ≥ 4 were considered to have positive expression, while those with scores < 4 were considered to be negative for the expression of FAM83A.

### Cell culture and transfection

The human cervical cancer cell lines HeLa and SiHa were purchased from the Shanghai Cell Bank (Shanghai, China) and cultured according to the instructions of the American Type Culture Collection (Manassas, VA, USA). HeLa and SiHa cells were cultured in Eagle's Minimum Essential Medium (Gibco, Invitrogen, NY, USA) at 37°C with 5% CO_2_. The cells were grown in 75-cm^2^ flasks, and the cell layer was briefly rinsed with 0.25% (w/v) trypsin0.53 mM EDTA solution to remove all traces of serum containing trypsin inhibitor every 1 or 2 days.

Cells were seeded in six-well plates for 24 h prior to transfection. The pCMV6-FAM83A plasmid and pCMV6 empty control vector were purchased from Origene (Rockville, MD, USA). Small interfering RNA (siRNA) against FAM83A (siFAM83A) and its control scrambled siRNA were purchased from RiboBio (Guangzhou, China). The plasmids or siRNAs were transfected into the cells using Lipofectamine® 3000 (Invitrogen, CA, USA) according to the manufacturer's instructions.

XAV-939, an inhibitor of the Wnt signaling pathway, was purchased from MedChemExpress (Monmouth Junction, NJ, USA). It was dissolved in dimethyl sulfoxide (Beijing Solarbio Science & Technology Co., Beijing, China) at a concentration of 20 nM. The inhibitor was added 24 h after transfection.

### Western blot assays

Western blot assays were performed as described previously 24. Briefly, total protein was extracted from the cells with cell lysis buffer (Pierce, Rockford, IL, USA) and quantified using the Bradford method. A total of 60 μg of protein was separated using 10% sodium dodecyl sulfate-polyacrylamide gel electrophoresis, followed by transfer to a polyvinylidene fluoride membrane (Millipore, Bedford, MA, USA). The membranes were blocked with 5% non-fat milk and incubated overnight at 4°C with primary antibodies. The antibodies used in this study are listed in Supplementary Table S1. Next, the membranes were incubated with horseradish peroxidase-conjugated mouse or rabbit IgG (1:2000, Proteintech, Wuhan, China) as appropriate for 2 h at 37°C. Protein bands were visualized with ECL (Pierce) and detected using the Bio Imaging System (DNR, Jerusalem, Israel). The relative protein levels were calculated using glyceraldehyde 3-phosphate dehydrogenase as a loading control.

### Cell proliferation and colony formation assays

Cell proliferation and colony formation assays were performed 24 h after transfection. For the cell proliferation assay, cells were cultured in a 96-well plate in medium containing 10% FBS. The cells were seeded at approximately 3000 cells/well, and the cell proliferation rate was determined using an MTS assay (Promega, Madison, WI, USA). MTS reagent (20 µL) was added to each well, and cells were incubated at 37°C. After 1 h, the cell proliferation rate was determined by measuring the absorbance of the cells at a wavelength of 490 nm on a spectrophotometer.

For the colony formation assay, cells were incubated on a 6-cm cell culture dish (800 cells/dish) for 14 days. The medium was changed every 4-5 days. Finally, the cells were fixed with 4% paraformaldehyde for 20 min and stained with hematoxylin for 30 min. Colonies with > 50 cells were counted.

### Matrigel invasion assay

Transwell chambers (Costar, Cambridge, MA, USA) with a pore size of 8 μm were used to detect the invasive ability of the cells. Matrigel (100 µL of a 1:7 dilution, BD Biosciences, CA, USA) was added to each insert, and the plates were placed in a 37°C incubator for at least 2 h. Twenty-four hours after transfection, 8 × 10^4^ cells were added to 100 μL medium supplemented with 2% FBS and seeded into the upper chamber. Medium supplemented with 20% FBS as the chemoattractant was added into the lower chamber. After incubation for 20 h, the non-invading cells on the top surface of the filters were swabbed with a cotton swab, and the cells adhering to the lower surface of the filters were fixed with 4% paraformaldehyde and stained with hematoxylin. Five high-power fields were selected randomly under a microscope to count the number of invasive cells.

### Immunofluorescence

Immunofluorescence assays were performed as described previously 24. Briefly, cells were fixed, permeabilized, and incubated with primary antibodies and fluorescein isothiocyanate-conjugated or tetramethylrhodamine isothiocyanate-conjugated secondary antibodies. Propidium iodide (50 μg/mL, Sigma, USA) was used to counterstain the nuclei. Finally, the cells were observed under a confocal microscope.

### Statistical analysis

All in vitro experiments were performed in triplicate. The statistical analyses were performed using the GraphPad Prism 6.0 statistical package (La Jolla, CA, USA). Potential correlations between FAM83A expression and clinicopathological factors were analyzed using the chi-squared test. All values in the in vitro experiments are expressed as mean ± standard deviation, and the differences between the groups were determined using one-way ANOVA multiple comparison test followed by Fisher's least significant difference test. Differences between groups were considered statistically significant at *P* < 0.05.

## Results

### FAM83A is highly expressed in cervical cancers and correlates with clinicopathological factors and poor patient prognosis

**Figure 1:**
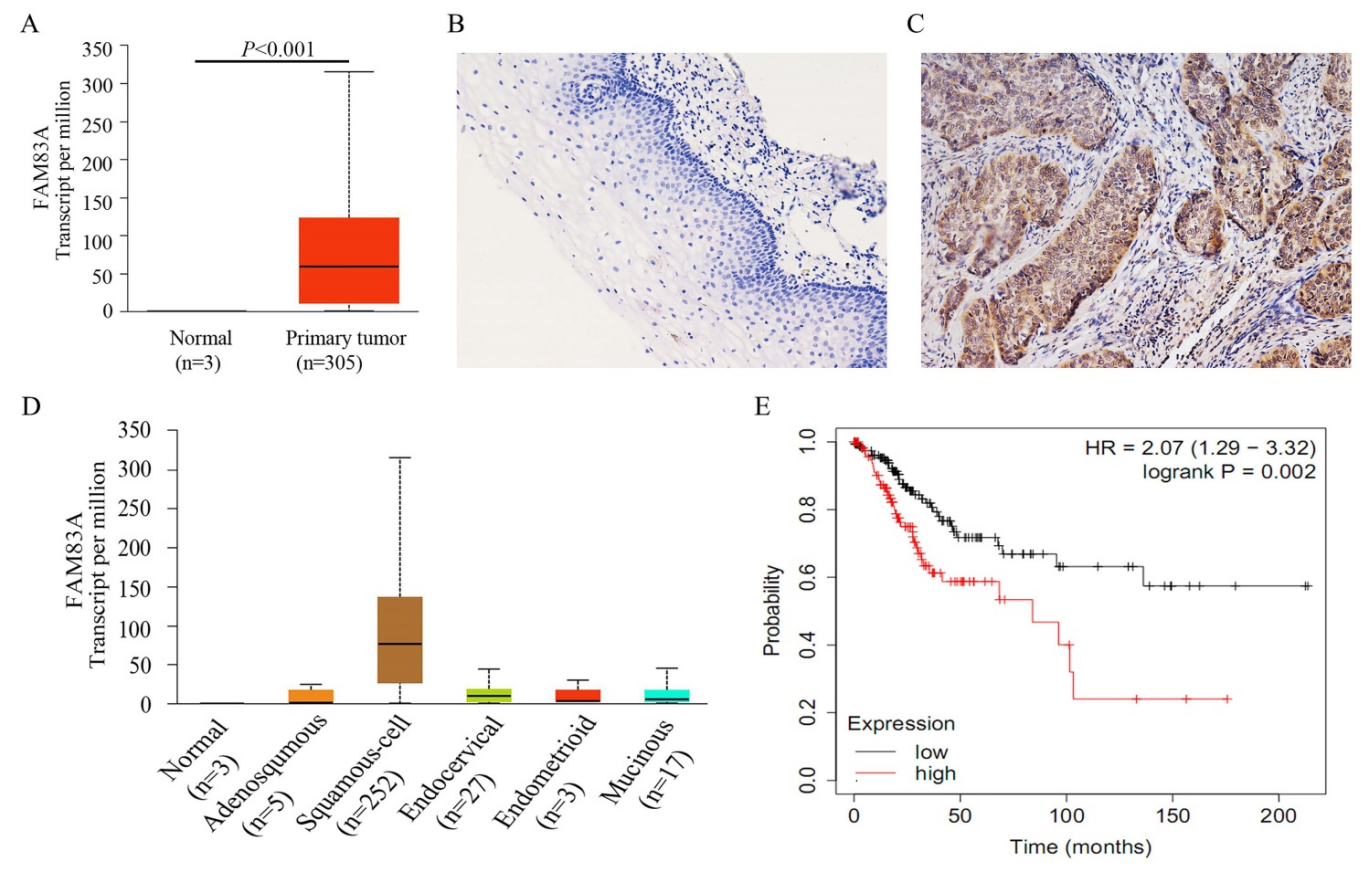
Expression of FAM83A in cervical cancer tissues. (A) Expression of FAM83A in cervical cancers and normal cervical tissues. data were downloaded from the UALCAN database. (B) Negative expression of FAM83A in normal cervical tissue. (Immunohistochemistry [streptavidin‐peroxidase method], original magnification ×200). (C) Positive expression of FAM83A in cervical squamous cell carcinoma tissue. (Immunohistochemistry [streptavidin‐peroxidase method], original magnification ×200). (D) Expression of FAM83A in the different histological subtypes of cervical cancers. data were downloaded from the UALCAN database. Adenosquamous: Cervical adenosquamous carcinoma; Squamous-cell: Cervical squamous cell carcinoma; Endocervical: Endocervical type of adenocarcinoma; Endometrioid: Endometrioid adenocarcinoma of endocervix; Mucinous: Mucinous adenocarcinoma of endocervical type. (E) Kaplan-Meier curves of cervical cancer patients by FAM83A expression. data were downloaded from the Kaplan‑Meier plotter database.

We analyzed the correlation between the expression of FAM83A and cervical cancer outcomes using UALCAN (http://ualcan.path.uab.edu) 21, which is a web-based tool for data mining. According to the data from UALCAN, FAM83A expression was much higher in cervical cancer tissues than in normal cervical tissues (*P* < 0.001) (Fig. 1A), suggesting that FAM83A may play a critical role in cervical cancer progression 25. CSCC had the highest FAM83A expression of all the histological subtypes of cervical cancer (*P* < 0.001) (Fig. 1D). According to Kaplan‑Meier analysis using the online database KM‑plotter 23, CSCC patients with high FAM83A expression had a shorter overall survival than patients with low FAM83A expression (*P* = 0.002; n = 304; median survival, 21.97 months for the high-expression cohort vs. 46.47 months for the low-expression cohort; Fig. 1E). In addition, based on the data downloaded from cBioPortal (http://www.cbioportal.org) 22, the mRNA level of *FAM83A* was positively correlated with *FAM83A* copy number alteration (correlation coefficient = 0.204, *P* < 0.001) and negatively correlated with its methylation status (correlation coefficient = -0.418, *P* < 0.001).

To confirm the results from online database, we examined the expression of FAM83A in 60 CSCCs and 45 corresponding normal cervical tissues. The positive rate of FAM83A in CSCCs (58.33%, 35/60) was significantly higher than that in normal cervical tissues (28.89%, 13/45; *P* = 0.005; Fig. 1B-C). As shown in Table 1, the expression of FAM83A protein was correlated with differentiation (*P* = 0.021), TNM stage (*P* = 0.006), and lymph node metastasis (*P* = 0.037), but not strongly correlated with patient age (*P* = 0.337) and tumor size (*P* = 0.326).

### FAM83A promotes proliferation, colony formation, and invasion in cervical cancer cells

**Figure 2:**
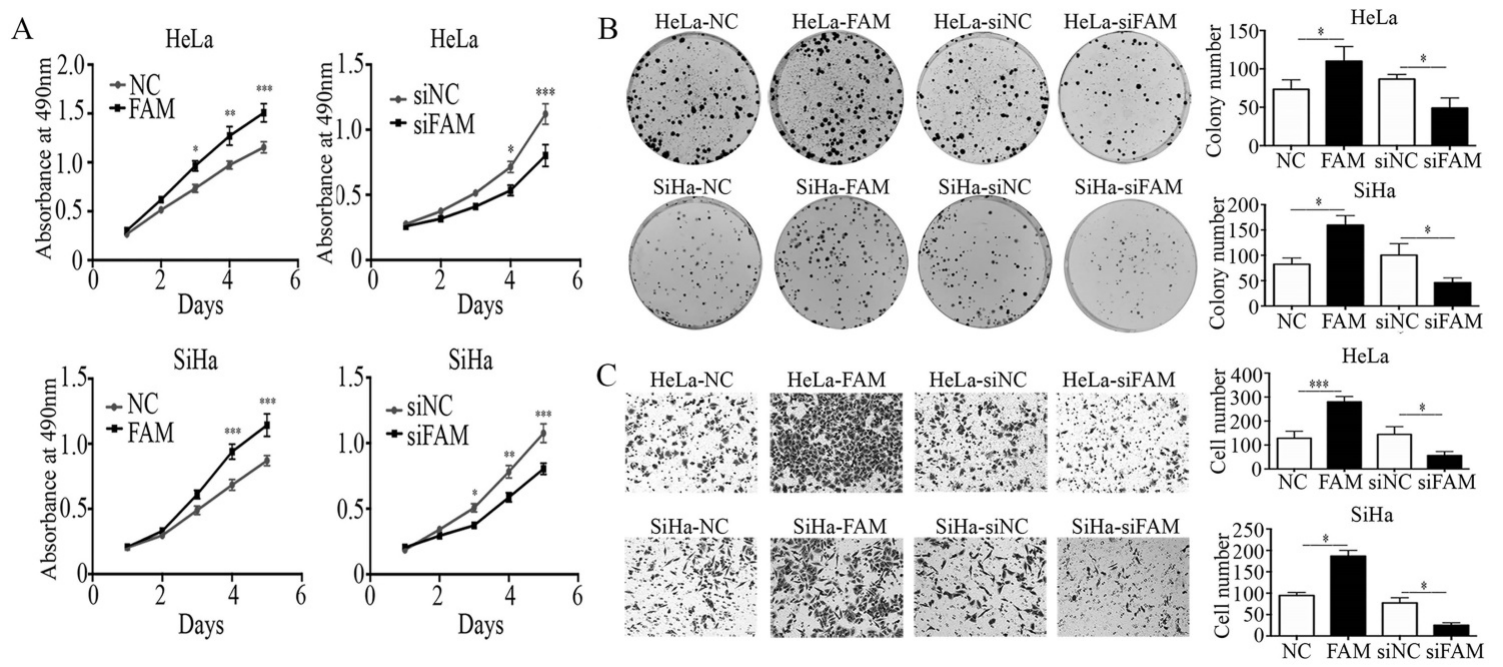
Effects of FAM83A on the proliferation, migration, and invasion of cervical cancer cells. (A) The growth curves of FAM83A-overexpressing or -knockdown cells and their respective control cells. (B) The numbers of colonies in FAM83A-overexpressing or -knockdown cells and control cells. The number of colonies formed by each cell type is shown in the histogram. (C) The Matrigel invasion assays of FAM83A-overexpressing or -knockdown cells and control cells. The invasive cell number of each type is shown in the histograms. NC, cells transfected with empty vector; FAM, cells transfected with *FAM83A*; siNC, cells transfected with scrambled siRNA. siFAM, cells interfered with siFAM83A. * *P* < 0.05, ** *P* < 0.01, *** *P* < 0.001.

To further investigate the effects of FAM83A on cell proliferation and invasion, we manipulated the expression of FAM83A in HeLa and SiHa cervical cancer cells. The overexpression or knockdown of FAM83A in these cells was confirmed at the protein level. The proliferative rates and number of colonies in cells transfected with *FAM83A* (HeLa-FAM83A and SiHa-FAM83A) were higher than those in the respective control cells (*P* < 0.05) (Fig. 2A-B). Matrigel invasion assays also demonstrated that the HeLa-FAM83A and SiHa-FAM83A cells had higher invasive abilities than the control cells (*P* < 0.05) (Fig. 2C). In contrast, after knocking down FAM83A expression in cervical cancer cells with siRNA (HeLa-siFAM83A and SiHa-siFAM83A), the proliferative rates, numbers of colonies, and invasive abilities were decreased (*P* < 0.05) (Fig. 2A-C).

### FAM83A regulates the expression of EMT-related proteins

**Figure 3:**
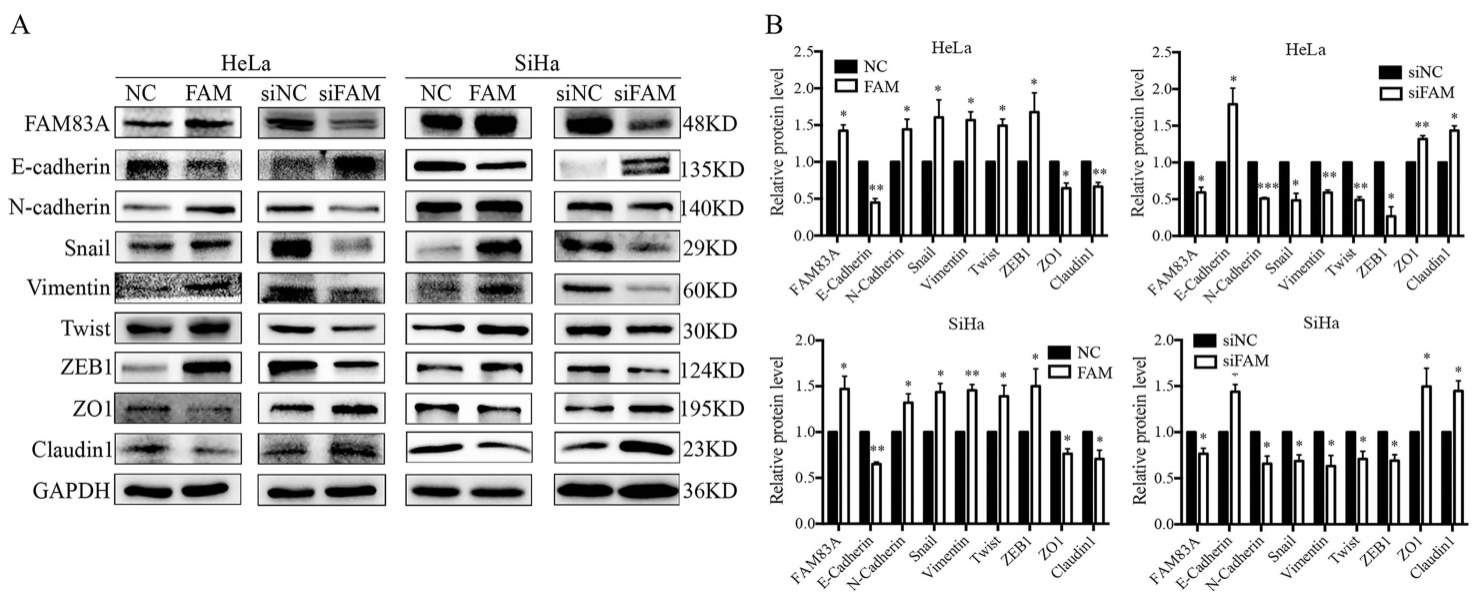
Effects of FAM83A on the expressions of epithelial-mesenchymal transition (EMT)-related proteins. (A) The expressions of EMT-related proteins in HeLa and SiHa cells. (B) Quantitation of the protein expression. Glyceraldehyde 3-phosphate dehydrogenase (GAPDH) served as an internal control. NC, cells transfected with empty vector; FAM, cells transfected with FAM83A; siNC: cells transfected with scrambled siRNA. siFAM, cells interfered with siFAM83A. * *P* < 0.05, ** *P* < 0.01, *** *P* < 0.001.

Next, we examined the expression of EMT-related proteins, including E-cadherin, N-cadherin, vimentin, ZEB1, twist, and snail, to explore the underlying mechanism by which FAM83A promotes the invasion of cervical cancer cells. The results showed that, after FAM83A transfection, the expression of E-cadherin was decreased, whereas the expressions of N-cadherin, vimentin, ZEB1, twist, and snail were significantly increased (*P* < 0.05) (Fig. 3). In contrast, after knocking down FAM83A, the expression of E-cadherin was increased, whereas the expressions of N-cadherin, vimentin, ZEB1, twist, and snail were significantly decreased (*P* < 0.05) (Fig. 3).

### FAM83A activates the Wnt signaling pathway

**Figure 4:**
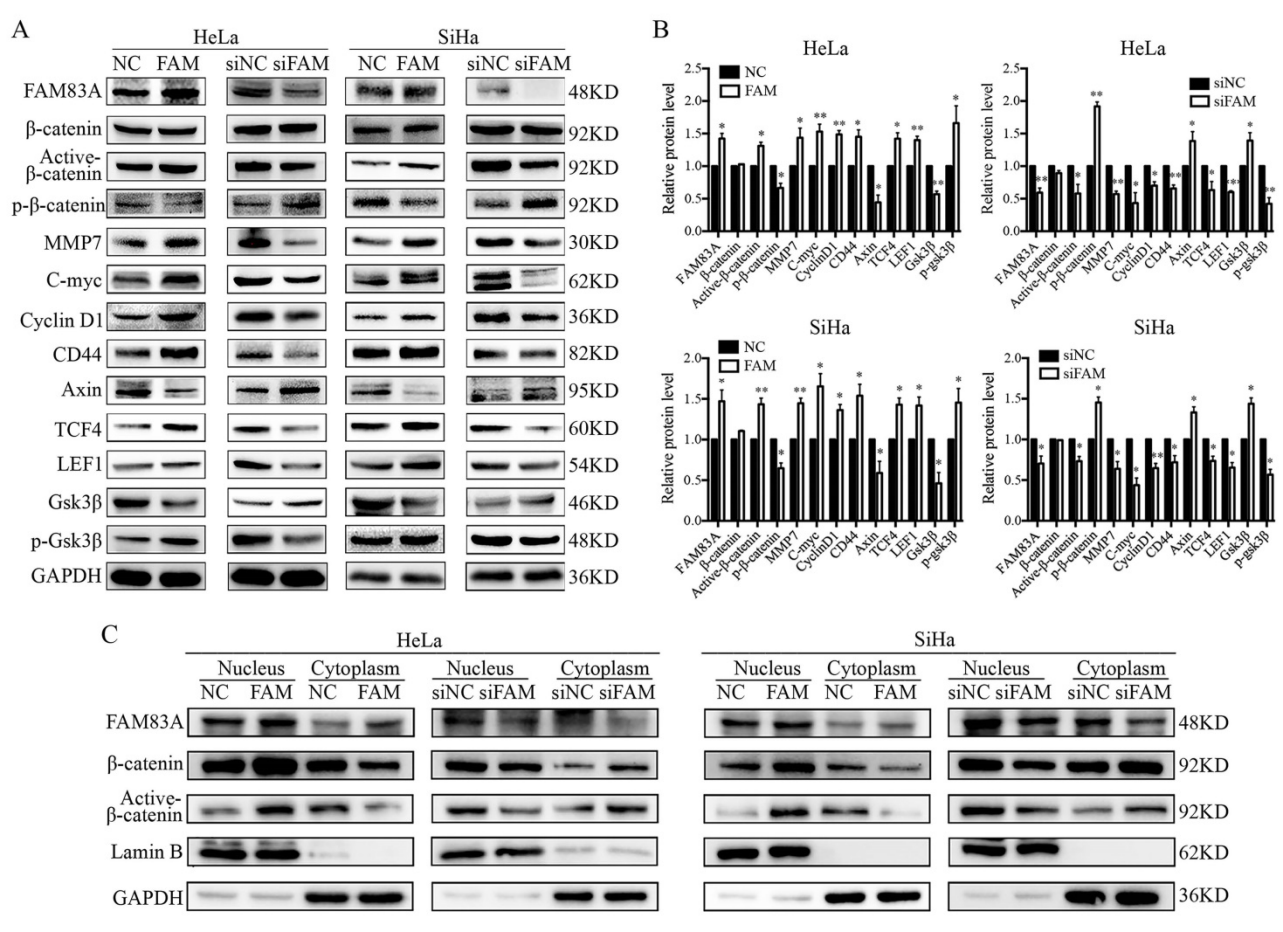
Effects of FAM83A on Wnt signaling pathway. (A) The expressions of key proteins in the Wnt signaling pathway. (B) Quantitation of the protein expression. Glyceraldehyde 3-phosphate dehydrogenase (GAPDH) served as an internal control. (C) The expressions of FAM83A, β-catenin, and active β-catenin in the cytoplasm and nucleus. GAPDH and Lamin B served as the internal cytoplasmic and nuclear controls, respectively. The relative protein levels in the control group were used to normalize protein levels in other groups. NC, cells transfected with empty vector; FAM, cells transfected with FAM83A; siNC, cells transfected with scrambled siRNA. siFAM, cells interfered with siFAM83A. * *P* < 0.05, ** *P* < 0.01, *** *P* < 0.001.

We investigated whether FAM83A regulated the activity of the Wnt signaling pathway. Compared with control cells, the expressions of key proteins in the Wnt signaling pathway, such as active β-catenin, TCF4, LEF1, and phosphorylated GSK3β, were increased, whereas the expressions of GSK3β, axin, and phosphorylated β-catenin were decreased in HeLa-FAM83A and SiHa-FAM83A cells (*P* < 0.05). The expressions of Wnt target genes, such as cyclin D1, c-Myc, MMP7, and CD44, were also increased after FAM83A transfection (*P* < 0.05) (Fig. 4A-B). However, the level of total β-catenin was not markedly changed. The opposite results were observed in HeLa-siFAM83A and SiHa-siFAM83A cells (*P* < 0.05) (Fig. 4A-B). To confirm these results, the nuclear and cytoplasmic levels of β-catenin and active β-catenin were examined. We found that the nuclear expressions of β-catenin and active β-catenin were both increased in HeLa-FAM83A and SiHa-FAM83A cells compared to those in control cells, whereas the cytoplasmic expressions of β-catenin and active β-catenin were both decreased (*P* < 0.05) (Fig. 4C). The opposite results were obtained after knocking down FAM83A in cervical cancer cells (*P* < 0.05) (Fig. 4C). Immunofluorescence experiments also confirmed these results (Supplementary Figure S1). These results indicated that FAM83A may facilitate the activation and nuclear translocation of β-catenin.

### FAM83A enhances the proliferative and invasive abilities of cervical cancer cells and regulates EMT via the Wnt signaling pathway

**Figure 5:**
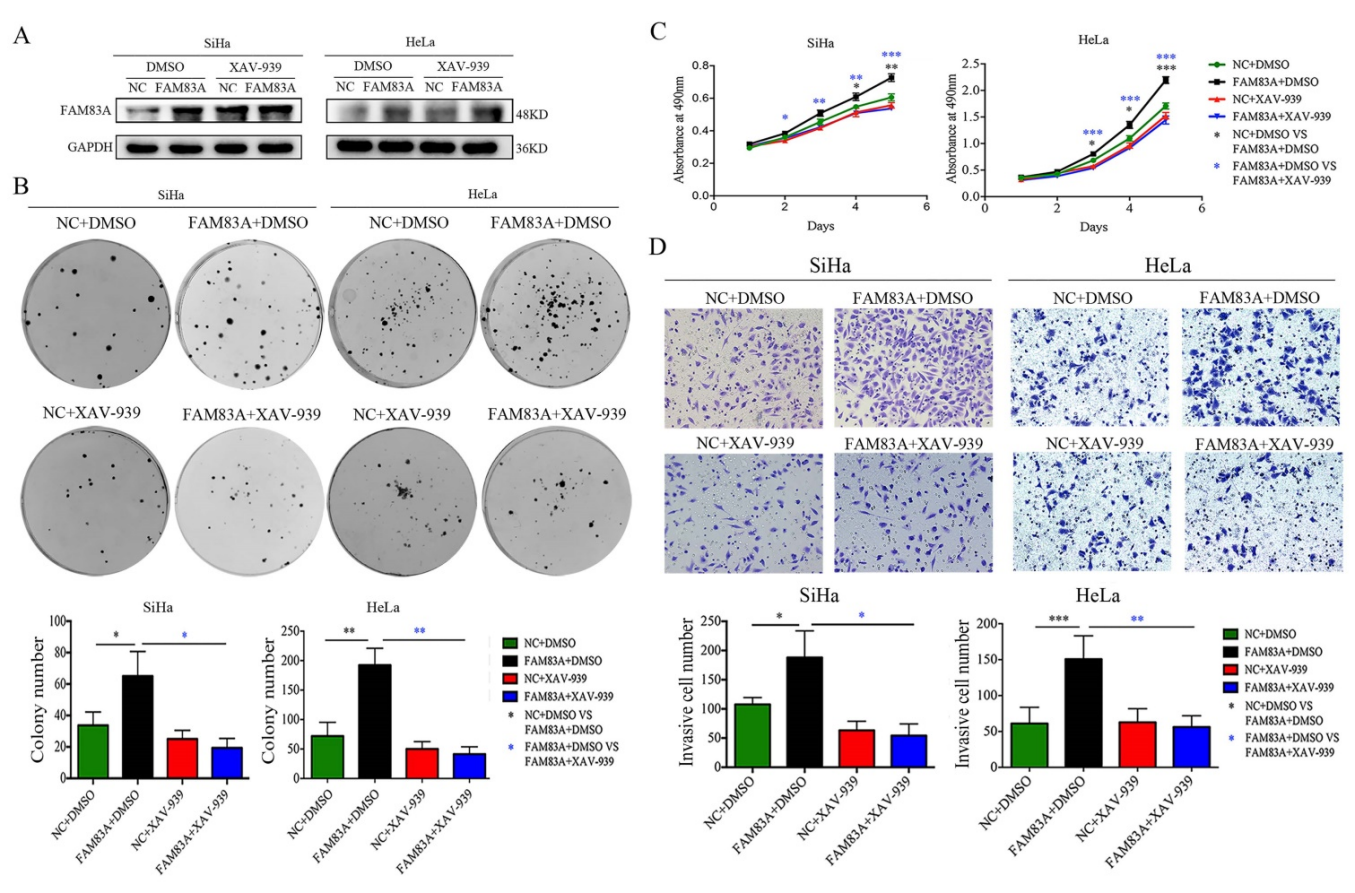
Effects of the Wnt inhibitor XAV-939 on the proliferation and invasion of FAM83A-overexpressing cervical cancer cells. (A) The levels of FAM83A in HeLa and SiHa cells transfected with *FAM83A* and treated with XAV-939. Glyceraldehyde 3-phosphate dehydrogenase (GAPDH) served as an internal control. (B-C) The numbers of colonies formed (B) and the growth curves (C) for HeLa and SiHa cells transfected with *FAM83A* and treated with XAV-939. (D) Images and quantitation of Matrigel invasion assays of HeLa and SiHa cells with or without FAM83A transfection and XAV-939 treatment. Cells treated with dimethyl sulfoxide (DMSO) served as a negative control. * *P* < 0.05, ** *P* < 0.01, *** *P* < 0.001.

**Figure 6:**
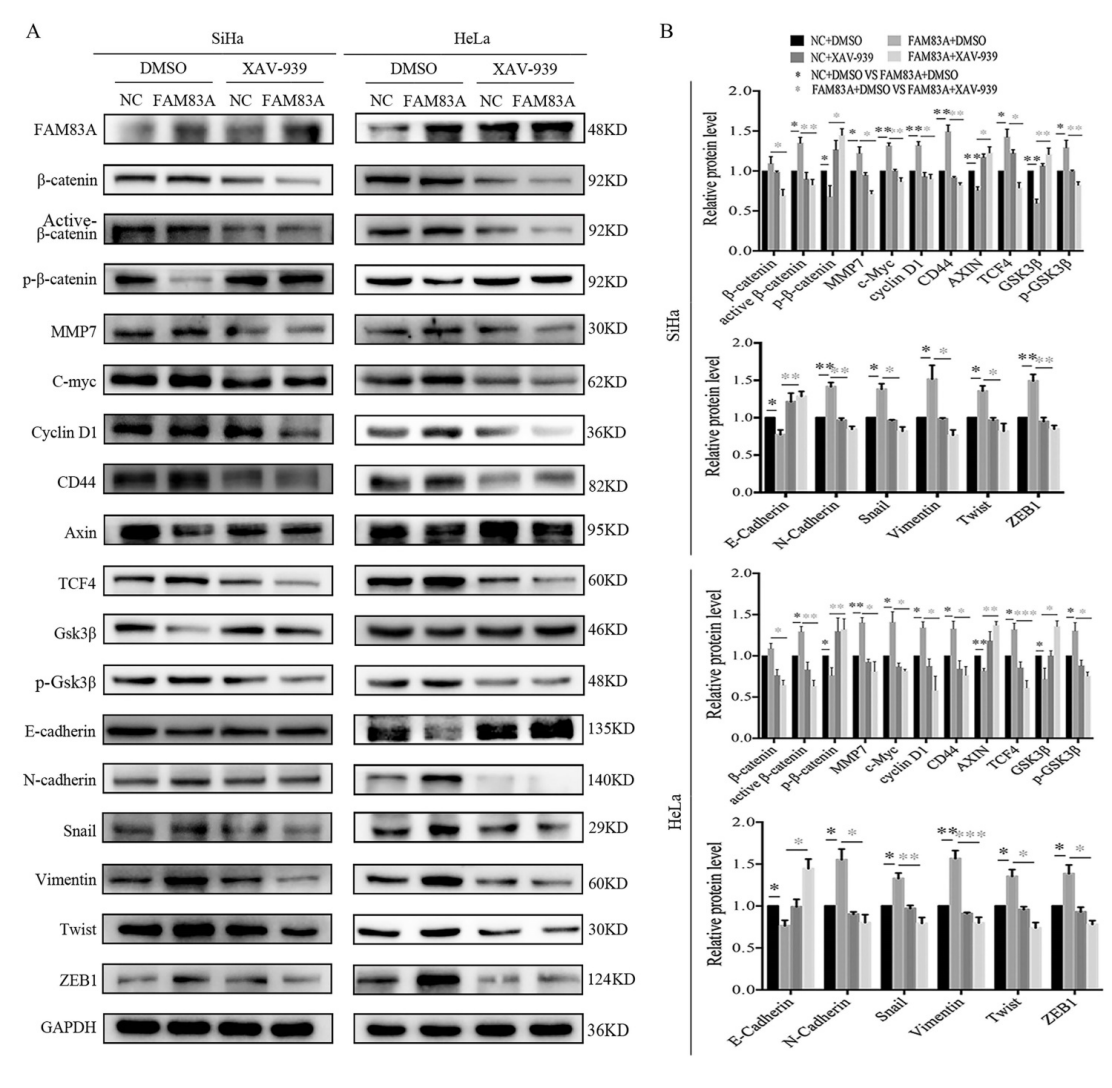
Effects of the Wnt inhibitor XAV-939 on the expressions of Wnt and epithelial-mesenchymal transition (EMT)-related proteins in FAM83A-overexpressing cervical cancer cells. (A) The expressions of Wnt and EMT-related proteins in HeLa and SiHa cells transfected with *FAM83A* and treated with XAV-939. Glyceraldehyde 3-phosphate dehydrogenase (GAPDH) served as an internal control. (B) Quantitation of the relative expressions of the proteins. The relative protein levels in the control group were used to normalize protein levels in other groups. Cells treated with dimethyl sulfoxide (DMSO) served as a negative control. * *P* < 0.05, ** *P* < 0.01, *** *P* < 0.001.

Cervical cancer cells were treated with the Wnt inhibitor XAV-939 to investigate whether FAM83A exerted its cancer-promoting role via the Wnt signaling pathway. As shown in Fig. 5, XAV-939 did not affect the expression of FAM83A. However, the increased proliferation, colony formation, and Matrigel invasion abilities induced by FAM83A overexpression in HeLa-FAM83A and SiHa-FAM83A cells were all reversed by XAV-939 (*P* < 0.05) (Fig. 5). Furthermore, the increased expressions of active β-catenin; Wnt target proteins, such as cyclin D1, c-Myc, MMP7, and CD44; and EMT-related proteins, such as N-cadherin, vimentin, ZEB1, twist, and snail, induced by FAM83A overexpression in HeLa-FAM83A and SiHa-FAM83A cells were also reversed by XAV-939 (*P* < 0.05) (Fig. 6).

## Discussion

The eight members of the FAM83 gene family are only present in vertebrates. They are defined by the presence of the conserved N-terminal DUF1669 domain, which includes a conserved phospholipase D-like catalytic motif 26. Many recent studies have indicated that FAM83A is a potential biomarker in multiple cancers, and this could provide a reference for treatment and predict the survival outcomes of patients with cancer 27, 28. FAM83A increases proliferation, invasion, drug resistance, and stem cell-like traits in breast, lung, hepatocellular, and pancreatic cancers 5, 9-12. However, the role of FAM83A in CSCC is insufficiently reported. Xu et al. revealed that FAM83A expression increased with cervical lesion progression from CIN to cervical cancer and that all lesions had higher levels of FAM83A than those in the normal cervix 14. The present study confirmed that FAM83A is highly expressed in cervical cancer tissues and is associated with the progression of CSCCs and poor patient prognosis. Our in vitro studies also confirmed that FAM83A promoted the proliferation and invasion of CSCC cells.

The mechanism by which FAM83A promotes cancer progression is not clear. Lee et al. showed that FAM83A confers epidermal growth factor receptor-tyrosine kinase inhibitor resistance in breast cancer cells and in mice using a distinct genetic screening for novel genes 12. FAM83A overexpression promotes invasion in hepatocellular carcinoma via the FAM83A/PI3K/AKT/c-Jun signaling pathway 11. Rong et al. also indicated that FAM83A is regulated by miR-206 and promotes cervical cancer progression through the PI3K/AKT/mTOR pathway 13. Liu et al. proposed that FAM83A is involved in the occurrence and development of lung cancer and is related to poor prognosis 29. Our previous study found that FAM83A can promote lung cancer progression by regulating the Wnt and Hippo signaling pathways 10. Several studies have shown that Wnt signaling pathway and EMT play important roles in the development of cervical cancer 20, 30-32. Therefore, we speculated that FAM83A promotes CSCC progression via Wnt signaling. The results of the present study showed that FAM83A promotes the activation of β-catenin and upregulates Wnt target genes, such as CD44, c-Myc*,* cyclin D1, and MMP7, and inhibits the expression and activation of GSK3β. In addition, overexpression of FAM83A promotes EMT. To confirm our results, we also verified the effects of FAM83A in cervical cancer CaSki cells (Supplementary Figure S2 and S3). Moreover, When CSCC cells were treated with XAV-939, an inhibitor of the Wnt pathway, the effects of FAM83A on cell proliferation and invasion were reversed. The activation of β-catenin, Wnt signaling, and EMT induced by FAM83A overexpression were also reversed by XAV-939 treatment. These results are consistent with our previous findings in lung cancer 10. Therefore, FAM83A may activate Wnt signaling and EMT through regulating the activity of β-catenin. However, the detailed mechanism needs further study. Recently, Xu et al. reported a result contradictory to the finding of their previous study 14, 15. They considered that FAM83A exerted a tumor-suppressive role in cervical cancer by regulating integrins 15. However, they could not explain why the expression of FAM83A is higher in cervical cancer tissues than in normal tissues. These contradictory results may be because of different study cohorts or because FAM83A is involved in different signaling pathways in different types of cancer cells. The role of FAM83A in cervical cancer still needs further confirmation.

In summary, FAM83A is highly expressed in cervical cancers and correlates with poor patient prognosis. FAM83A promotes the proliferative and invasive abilities of CSCC cells by regulating Wnt signaling pathway and EMT.

## Supplementary Material

Supplementary figures and table.

## Figures and Tables

**Table 1:** Correlations between FAM83A expression and clinicopathological factors in cervical squamous cell carcinomas.

	n	FAM83A-positive	FAM83A-negative	*P*-value
**Tissues**				0.005
Normal cervical tissue	45	13	32	
Cervical squamous cell carcinoma	60	35	25	
**Age**				0.337
< 60	34	19	15	
≥ 60	26	16	10	
Differentiation				0.021
Well	19	10	9	
Moderate-Poor	41	25	16	
**TNM stages**				0.006
I	14	6	8	
II	23	13	10	
III	23	16	7	
Tumor size				0.326
≤ 2 cm	43	25	18	
> 2 cm	17	10	7	
Lymphatic metastasis				0.037
No	39	21	18	
Yes	21	14	7	

## Abbreviations

FAM83A: family with sequence similarity 83, member A
CIN: cervical intraepithelial neoplasia
EMT: epithelial-mesenchymal transition
CSCC: cervical squamous cell carcinoma
siRNA: small interfering RNA
TCGA: The Cancer Genome Atlas

## References

[1] Bray F, Ferlay J, Soerjomataram I, Siegel RL, Torre LA, Jemal A (2018). Global cancer statistics 2018: GLOBOCAN estimates of incidence and mortality worldwide for 36 cancers in 185 countries. CA Cancer J Clin.

[2] The L (2020). Eliminating cervical cancer. Lancet (London, England).

[3] Lee KB, Kim YS, Lee JM (2018). Oncologic outcomes of adjuvant chemotherapy alone after radical surgery for stage IB-IIA cervical cancer patients. J Gynecol Oncol.

[4] Ehrke-Schulz E, Heinemann S, Schulte L, Schiwon M, Ehrhardt A (2020). Adenoviral Vectors Armed with PAPILLOMAVIRUs Oncogene Specific CRISPR/Cas9 Kill Human-Papillomavirus-Induced Cervical Cancer Cells. Cancers (Basel).

[5] Zhou F, Geng J, Xu S, Meng Q, Chen K, Liu F (2019). FAM83A signaling induces epithelial-mesenchymal transition by the PI3K/AKT/Snail pathway in NSCLC. Aging (Albany NY).

[6] Fulcher LJ, Bozatzi P, Tachie-Menson T, Wu KZL, Cummins TD, Bufton JC (2018). The DUF1669 domain of FAM83 family proteins anchor casein kinase 1 isoforms. Sci Signal.

[7] Bartel CA, Parameswaran N, Cipriano R, Jackson MW (2016). FAM83 proteins: Fostering new interactions to drive oncogenic signaling and therapeutic resistance. Oncotarget.

[8] Li Y, Dong X, Yin Y, Su Y, Xu Q, Zhang Y (2005). BJ-TSA-9, a novel human tumor-specific gene, has potential as a biomarker of lung cancer. Neoplasia.

[9] Chen S, Huang J, Liu Z, Liang Q, Zhang N, Jin Y (2017). FAM83A is amplified and promotes cancer stem cell-like traits and chemoresistance in pancreatic cancer. Oncogenesis.

[10] Zheng YW, Li ZH, Lei L, Liu CC, Wang Z, Fei LR (2020). FAM83A Promotes Lung Cancer Progression by Regulating the Wnt and Hippo Signaling Pathways and Indicates Poor Prognosis. Front Oncol.

[11] Liu C, Peng X, Li Y, Liu S, Hou R, Zhang Y (2020). Positive feedback loop of FAM83A/PI3K/AKT/c-Jun induces migration, invasion and metastasis in hepatocellular carcinoma. Biomed Pharmacother.

[12] Lee SY, Meier R, Furuta S, Lenburg ME, Kenny PA, Xu R (2012). FAM83A confers EGFR-TKI resistance in breast cancer cells and in mice. J Clin Invest.

[13] Rong L, Li H, Li Z, Ouyang J, Ma Y, Song F (2020). FAM83A as a Potential Biological Marker Is Regulated by miR-206 to Promote Cervical Cancer Progression Through PI3K/AKT/mTOR Pathway. Front Med (Lausanne).

[14] Xu J, Liu H, Yang Y, Wang X, Liu P, Li Y (2019). Genome-Wide Profiling of Cervical RNA-Binding Proteins Identifies Human Papillomavirus Regulation of RNASEH2A Expression by Viral E7 and E2F1. mBio.

[15] Xu J, Lu W (2020). FAM83A exerts tumor-suppressive roles in cervical cancer by regulating integrins. Int J Oncol.

[16] van Schie EH, van Amerongen R (2020). Aberrant WNT/CTNNB1 Signaling as a Therapeutic Target in Human Breast Cancer: Weighing the Evidence. Front Cell Dev Biol.

[17] Lin J, Song T, Li C, Mao W (2020). GSK-3β in DNA repair, apoptosis, and resistance of chemotherapy, radiotherapy of cancer. Biochim Biophys Acta Mol Cell Res.

[18] Wang Z, Li Z, Wu Q, Li C, Li J, Zhang Y (2020). DNER promotes epithelial-mesenchymal transition and prevents chemosensitivity through the Wnt/β-catenin pathway in breast cancer. Cell Death Dis.

[19] Dong L, Dong Q, Chen Y, Li Y, Zhang B, Zhou F (2018). Novel HDAC5-interacting motifs of Tbx3 are essential for the suppression of E-cadherin expression and for the promotion of metastasis in hepatocellular carcinoma. Signal Transduct Target Ther.

[20] Münsterberg J, Loreth D, Brylka L, Werner S, Karbanova J, Gandrass M (2020). ALCAM contributes to brain metastasis formation in non-small-cell lung cancer through interaction with the vascular endothelium. Neuro Oncol.

[21] Chandrashekar DS, Bashel B, Balasubramanya SAH, Creighton CJ, Ponce-Rodriguez I, Chakravarthi B (2017). UALCAN: A Portal for Facilitating Tumor Subgroup Gene Expression and Survival Analyses. Neoplasia (New York, NY).

[22] Gao J, Aksoy BA, Dogrusoz U, Dresdner G, Gross B, Sumer SO (2013). Integrative analysis of complex cancer genomics and clinical profiles using the cBioPortal. Sci Signal.

[23] Nagy A, Munkacsy G, Gyorffy B (2021). Pancancer survival analysis of cancer hallmark genes. Sci Rep.

[24] Fei LR, Huang WJ, Wang Y, Lei L, Li ZH, Zheng YW (2019). PRDM16 functions as a suppressor of lung adenocarcinoma metastasis. J Exp Clin Cancer Res.

[25] Zheng H, Zhang G, Zhang L, Wang Q, Li H, Han Y (2020). Comprehensive Review of Web Servers and Bioinformatics Tools for Cancer Prognosis Analysis. Front Oncol.

[26] Bozatzi P, Sapkota GP (2018). The FAM83 family of proteins: from pseudo-PLDs to anchors for CK1 isoforms. Biochem Soc Trans.

[27] Richtmann S, Wilkens D, Warth A, Lasitschka F, Winter H, Christopoulos P (2019). FAM83A and FAM83B as Prognostic Biomarkers and Potential New Therapeutic Targets in NSCLC. Cancers.

[28] Grant S (2012). FAM83A and FAM83B: candidate oncogenes and TKI resistance mediators. J Clin Invest.

[29] Liu L, Liao GQ, He P, Zhu H, Liu PH, Qu YM (2008). Detection of circulating cancer cells in lung cancer patients with a panel of marker genes. Biochem Biophys Res Commun.

[30] Wang B, Li X, Liu L, Wang M (2020). β-Catenin: oncogenic role and therapeutic target in cervical cancer. Biol Res.

[31] Hu M, Guo W, Liao Y, Xu D, Sun B, Song H (2019). Dysregulated ENPP1 increases the malignancy of human lung cancer by inducing epithelial-mesenchymal transition phenotypes and stem cell features. Am J Cancer Res.

[32] Ahmad A, Ansari IA (2020). A Comprehensive Review on Crosstalk of Human Papilloma Virus oncoproteins and developmental/self-renewal pathways during the pathogenesis of uterine cervical cancer. Curr Mol Med.

