# Supplementary material for: FAM83A Promotes the Proliferative and Invasive Abilities of Cervical Cancer Cells via Epithelial-Mesenchymal Transition and the Wnt Signaling Pathway
Source: J Cancer. 2021 Aug 28;12(21):6320–9. doi: 10.7150/jca.62563 (PMC8489145; doi:10.7150/jca.62563)

Table S1 Antibodies description

Antibodies names	Dilution concentration	Brand and origin
FAM83A	1:1000	Proteintech, Wuhan, China
β-catenin	1:1000	Cell Signaling Technology, MA, USA
active β-catenin	1:1000	Cell Signaling Technology, MA, USA
p-β-catenin	1:1000	Proteintech, Wuhan, China
GSK3β	1:1000	Cell Signaling Technology, MA, USA
p-GSK3β	1:1000	Proteintech, Wuhan, China
cyclinD1	1:1000	Proteintech, Wuhan, China
MMP7	1:1000	Proteintech, Wuhan, China
c-Myc	1:1000	Proteintech, Wuhan, China
CD44	1:1000	Proteintech, Wuhan, China
TCF4	1:1000	Proteintech, Wuhan, China
LEF1	1:200	Santa Cruz Biotechnology, CA, USA
Axin	1:200	Santa Cruz Biotechnology, CA, USA
E-cadherin	1:1000	Cell Signaling Technology, MA, USA
N-cadherin	1:1000	Cell Signaling Technology, MA, USA
Vimentin	1:1000	Proteintech, Wuhan, China
Snail	1:500	Proteintech, Wuhan, China
ZEB1	1:1000	Proteintech, Wuhan, China
ZO-1	1:1000	Proteintech, Wuhan, China
Twist	1:1000	Proteintech, Wuhan, China
HRP-conjugated Affinipure Goat Anti-Mouse IgG(H+L)	1:1000	Proteintech, Wuhan, China
Claudin1	1:1000	Proteintech, Wuhan, China
LaminB	1:1000	Cell Signaling Technology, MA, USA
SignalStain Boost IHC Detection Reagent (HRP, Rabbit)	1:1000	Cell Signaling Technology, MA, USA

Figure S1 Immunofluorescence showing the expression and localization of β-catenin and active β-catenin in the nucleus and cytoplasm.

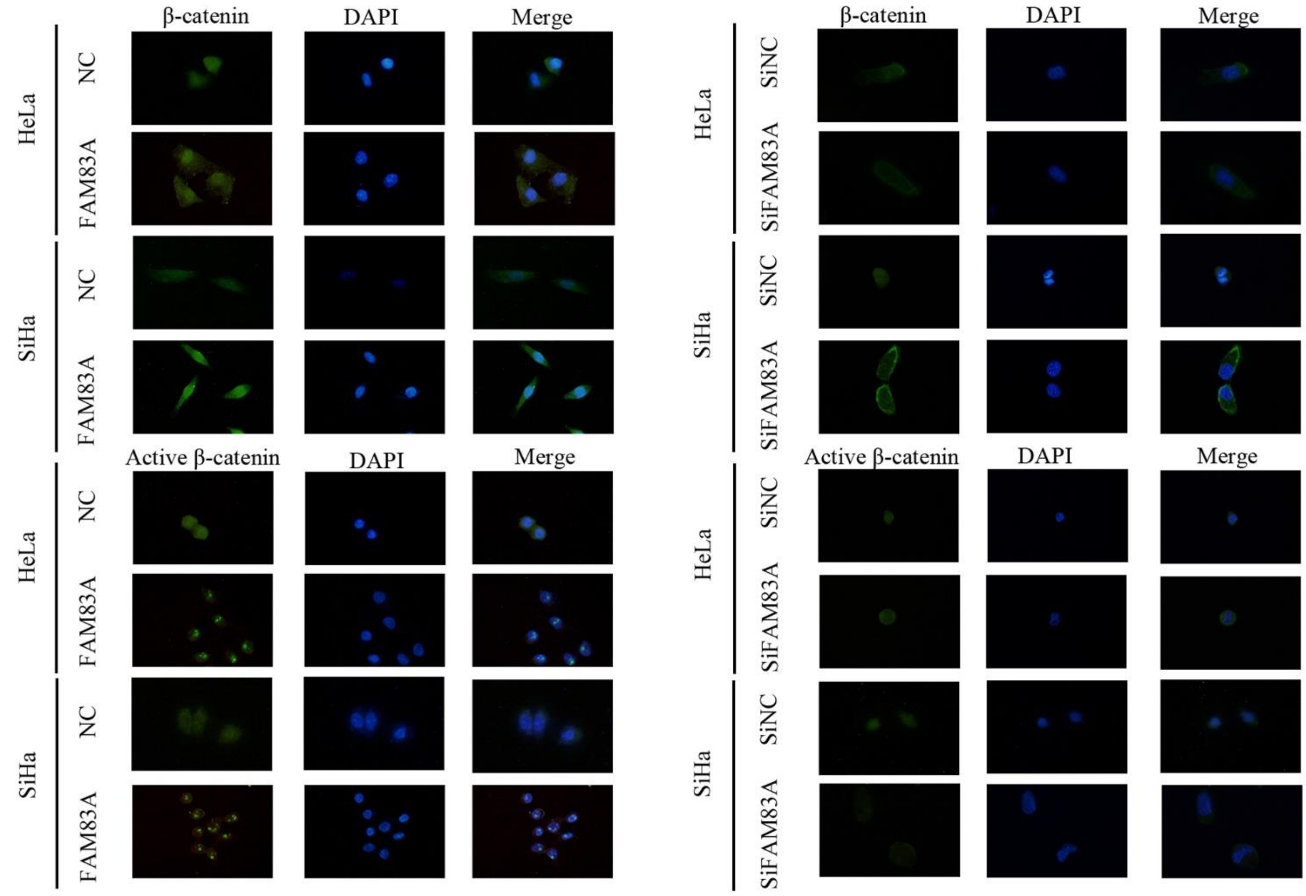

Figure S2 Effects of FAM83A on the proliferation, migration, and invasion of cervical cancer CaSki cells.

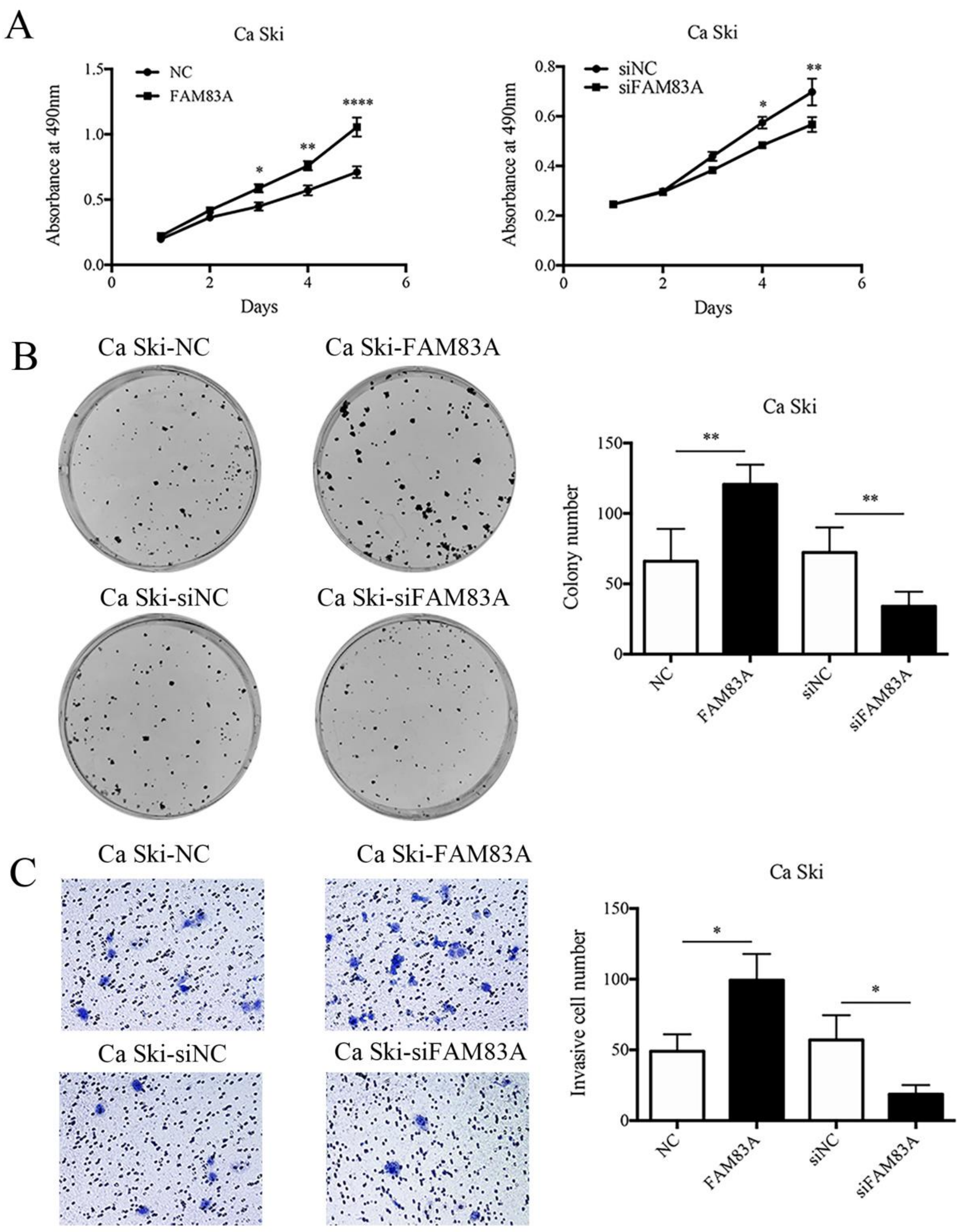

Figure S3 Effects of FAM83A on the expressions of Wnt signaling pathway and epithelial–mesenchymal transition (EMT)-related proteins in CaSki cells.

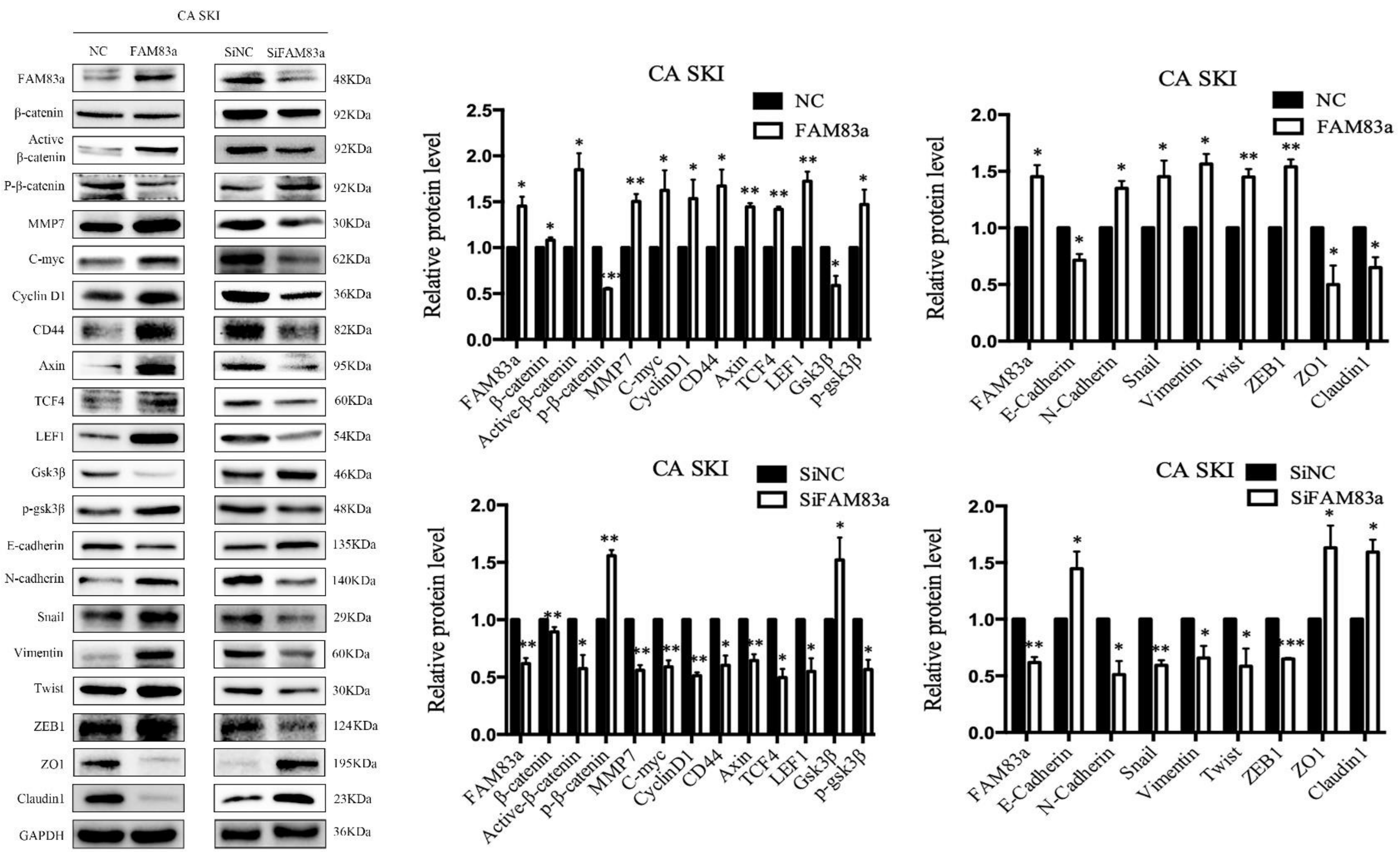

Supplement: Supplementary file 1 — Supplementary figures and table. [file jcav12p6320s1.pdf]
